# Doping LiFePO_4_ with Al^3+^: Suppression of Anti-Site Defects and Implications for Battery Recycling

**DOI:** 10.1021/acsomega.4c08870

**Published:** 2025-01-06

**Authors:** Yunhao Xiao, Zihang Zhao, Qipeng Zhang, Rui Qiao

**Affiliations:** Department of Mechanical Engineering, Virginia Tech, Blacksburg, 635 Prices Fork Road, Blacksburg, Virginia 24061, United States

## Abstract

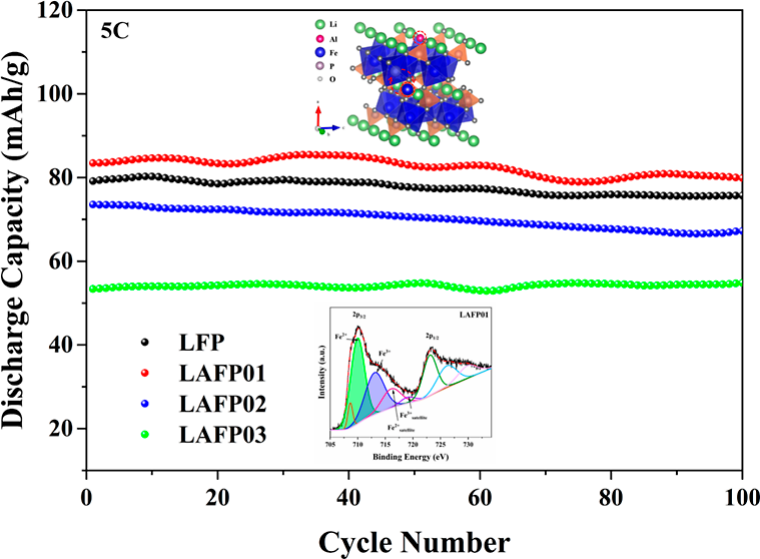

In this study, a group of aluminum-doped lithium iron phosphate (LFP) with varying dopant concentrations (Li_1–3*x*_Al_*x*_FePO_4_/C, where *x* = 0.01–0.03) was synthesized via a solid-state reaction. Comprehensive analysis revealed that the aluminum dopant was uniformly distributed across the crystals of the synthesized samples. Notably, minor doping (*x* ≤ 0.01) helped reduce the formation of antisite defects within the LFP structure, lowering the defect content to 1.67% compared to 2.04% in undoped LFP. Further examination corroborated the presence of antisite defects and confirmed their reduced concentration in aluminum-doped LFP. Electrochemically, LAFP01 with *x* = 0.01 (or 1% aluminum doping) demonstrated an increased lithium-ion diffusion coefficient and superior electrochemical performance, achieving a discharge capacity of 155.6 mA h/g at a 0.1 C rate and surpassing that of undoped LFP. The performance improvement was more evident under rapid charge and discharge conditions, where LAFP01 maintained a higher specific capacity (86 mA h/g compared to 78 mA h/g for undoped LFP) at a current density of 5 C or greater. This study suggests that the reduced antisite defects with small aluminum doping could potentially contribute to the improved electrochemical characteristics of LFP cathodes, offering insights into enhancing lithium-ion battery performance and managing aluminum impurities in battery recycling processes.

## 1 Introduction

Lithium iron phosphate (LiFePO_4_), a cathode material for lithium-ion batteries, has been studied extensively over the past decades.^1^ Its distinct attributes, including cost-effectiveness, high power density, excellent long-term cycling stability, and exceptional safety, have led to a surge in demand in rapidly expanding industries such as electric vehicles and battery electric storage systems.^2^ This demand is anticipated to continue accelerating. However, the widespread application of LIBs also presents challenges related to material utilization and environmental impact, making it a key focus for researchers and engineers to find solutions.

First of all, due to the inherent limitations of electrodes, including low electronic conductivity and restricted Li^+^ ion diffusion rate,^3^ the potential of LiFePO_4_ is still not fully unleashed. In manufacturing lithium-ion cathodes, significant efforts have been made to overcome those limitations, including (1) coating a thin layer of carbon to improve the apparent conductivity;^4^ (2) reducing particle size to shorten the Li-ion diffusing path inside particles, thereby boosting the diffusion rate;^5^ and (3) doping with transition metals such as Ce, Mg, Mn, Nb, Al, and V into M1, M2, or both sites in LFP to alter the [010] channel and increase the Li^+^ diffusion rate.^6−10^ Depending on the specific requirement, the mentioned strategies can be applied individually or in combination. However, the first two methods have a more significant impact on the product’s physical properties. For example, changing carbon coating affects the total percentage of the active material in a single battery pack, and adjusting the electrode’s particle size alters particle agglomeration and overall powder tape density, which can greatly influence the gravimetric and volumetric energy of batteries. Therefore, the adjustable range of these parameters must be narrow to ensure an acceptable and deliverable specific energy for the battery. In contrast, doping elements in the LiFePO_4_ material can result in similar or even more pronounced improvements in cathode electrochemistry while only altering the material at the angstrom level, making it a preferred solution for LFP modification. In particular, aluminum, compared to other dopants, is inexpensive and easy to incorporate into the LFP, highlighting its potential research value.

Second, given the average service life of 5–8 years for current lithium-ion batteries, the effective and sustainable disposal of end-of-life batteries is crucial. Consequently, in the realm of battery recycling, strategies such as element recovery through pyrometallurgy and hydrometallurgy as well as direct recycling have been developed to optimize the utilization of end-of-life LIBs. Regardless of the recycling routes, the removal of aluminum, which has been used as the cathode current collector since the introduction of the first commercial Sony LIB in 1991,^11^ is essential. However, despite careful processing, aluminum (Al) is still retained in the recovered material, leading to the likely introduction of some aluminum into regenerated LiFePO_4_.

Whether the primary focus is on understanding the influence of aluminum (Al) on fresh lithium iron phosphate (LFP) materials or on estimating the potential impact of residual aluminum impurities in regenerated LFP, an extensive study of the effects of Al-doping on LFP is crucial. Research has shown that the Al^3+^ ions can be doped at both the Li site (M1) and Fe site (M1) in LFP.^12,13^ Kulka *et al*. evaluated the formation of Li(Al)FePO_4_ and demonstrated that the partial substitution of Li by Al enhances the material’s performance, attributing this improvement to the creation of Li vacancies resulting from a stoichiometric deficiency of Li.^9^ Similarly, Amin *et al*. synthesized LFP with Al-doped at the Fe site and investigated its anisotropic conductivity. They suggested that the increased ionic conductivity in LiFe(Al)PO_4_ could be explained by vacancy association with the impurity.^14,15^ Although antisite defects, a common type of structural disorder similar to Li vacancies and holes in crystals, have been widely discussed in the literature, no definitive evidence has yet ruled out their relevance to the performance changes observed in these samples. Furthermore, detailed investigations into antisite defects in Al-doped LiFePO_4_ remain limited.

In this study, a series of LFP with varying aluminum (Al) doping contents on the Li-site were synthesized via a one-step solid-state process.^16^ The structural changes in LFP induced by aluminum doping, particularly focusing on the Fe_Li_^·^ antisite defect (in Kroger–Vink notation), were systematically investigated using a combination of analytical techniques, including X-ray Diffraction with Rietveld refinement (XRD), Scanning Electron Microscopy (SEM), Fourier-Transform Infrared Spectroscopy (FT-IR), X-ray Photoelectron Spectroscopy (XPS), and comprehensive electrochemical analysis.

The findings suggest that an appropriate amount of Al^3+^ ions may inhibit the formation of Fe_Li_^·^ antisite defects, potentially facilitating smoother migration along the [010] channel for Li-ion intercalation and deintercalation. This enhancement leads to a higher discharge capacity in Al-doped LiFePO_4_ compared to that of its undoped counterpart. Additionally, as the current density increases, the blocking effect of Fe_Li_^·^ antisite defects on the electrochemical performance of LFP becomes more pronounced, highlighting the benefits of optimal Al doping.

A key contribution of this study is the comprehensive investigation of antisite defect suppression via Al doping, proposing this suppression as a potential mechanism for the observed performance improvements in LFP. Unlike previous studies, we quantitatively assessed the relationship between Al doping concentration and antisite defect formation, identifying an optimal doping range for enhancing electrochemical properties. These findings could also guide cost optimization strategies in battery recycling by providing practical recommendations for managing Al impurities on an industrial scale.

## 2 Experimental Section

### 2.1 Sample Preparation

The undoped and aluminum-doped lithium iron phosphate (LAFP/C) were synthesized through a one-step solid-state method by using lithium carbonate (Li_2_CO_3_), iron oxalate dihydrate (FeC_2_O_4_·2H_2_O), and ammonium dihydrogen phosphate (NH_4_H_2_PO_4_) as raw materials, while glucose served as a carbon coating precursor, and aluminum acetylacetonate (Al(C_5_H_7_O_2_)_3_) was utilized as the doping agent. The molar ratio of aluminum was systematically increased from 0.01 in LAFP01 (1% Al doping) to 0.03 in LAFP03 (3% Al doping). The mixtures were ball-milled in ethanol for 48 h under an inert atmosphere and then dried in a vacuum oven at 60 °C for 12 h. The resulting powders were then preheated at 350 °C for 10 h followed by sintering at 700 °C for an additional 20 h with continuous argon flow. The final products were named LFP for the undoped sample and LAFP01, LAFP02, and LAFP03 for the Al-doped LiFePO_4_/C, corresponding to Al contents of 1% to 3%.

### 2.2 Instrumental Analysis

The elemental composition, notably Al concentration, of the synthesized samples was initially determined using a Thermo Electron iCAP RQ ICP-MS. Carbon coating content was investigated with a TA-TGA 550 (thermogravimetric analyzer). Lattice stripes and carbon coating uniformity and thickness were characterized by transmission electron microscopy (JEOL JEM-2100). Sample morphologies and elemental distributions were analyzed by using a JEOL IT-500HR scanning electron microscope with an EDS mapping feature. Phase composition and crystal structure were investigated by using a Rigaku MiniFlex X-ray diffractometer, acquired at a wavelength of 1.5406 Å (Cu Kα), employing Rietveld refinement to obtain detailed lattice information and site occupancies. Vibrational analysis was conducted through Fourier transform infrared spectroscopy (FT-IR), and XPS spectra for Fe 2p were obtained by using a PHI Quantera SXM.

### 2.3 Electrochemical Performance Measurement

The cathode for electrochemical tests was fabricated by mixing the active material, Super P, and poly(vinylidene fluoride) (PVDF) in a 1-methyl-2-pyrrolidinone (NMP) solvent at a mass ratio of 80:10:10. The resulting slurry was cast onto an Al foil and vacuum-dried for 8 h at 105 °C, with a mass loading of the dried electrodes of 2.4 mg/cm^2^. Subsequently, the cathode was assembled in a 2032-type coin cell inside an argon-filled glovebox with lithium foil as the counter electrode and Celgard 2400 as the separator. The electrolyte consisted of 1 M LiPF_6_ in a 50/50 ethylene carbonate/dimethyl carbonate (EC/DMC) mixture with 10 v % fluoroethylene carbonate (FEC). Electrochemical tests, including rate performance and long-term cycling, were conducted at room temperature using a Land CT2001 tester, and electrochemical impedance spectroscopy (EIS) was carried out with a Biologic SP-150 Potentiostat over a frequency range of 100 kHz to 10 mHz.

## 3 Results and Discussion

### 3.1 Morphology and Structural Analysis

#### 3.1.1 Scanning Electron Microscopy and Transmission Electron Microscopy

**Figure 1:**
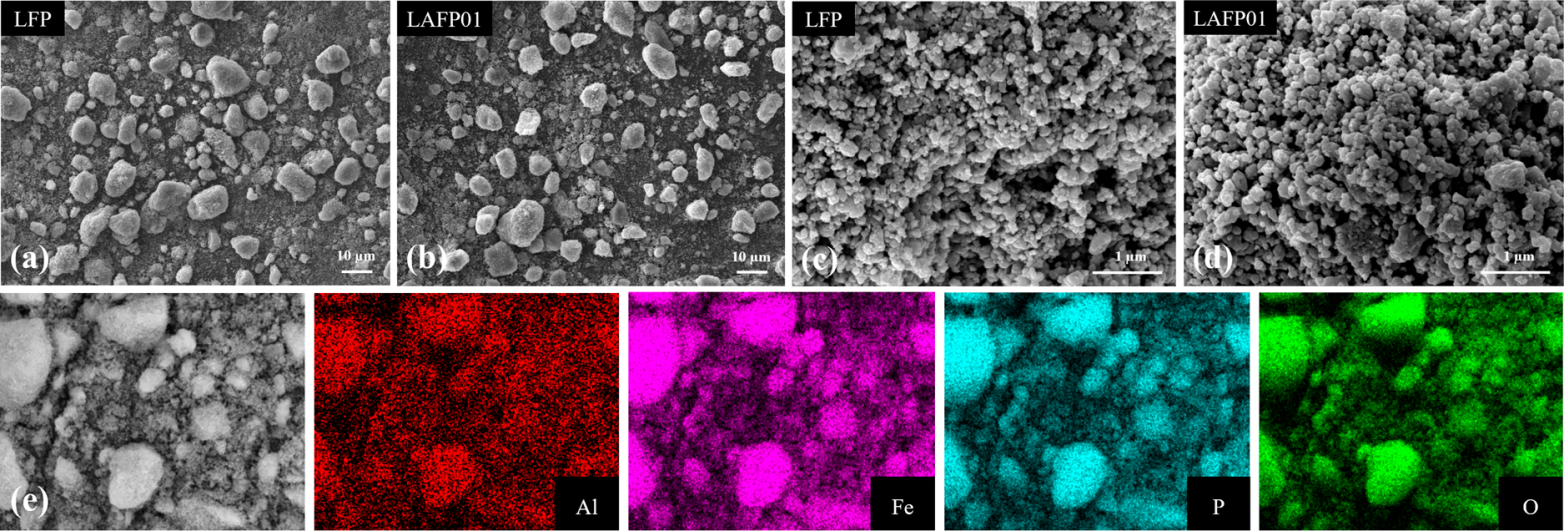
SEM images of LFP/C (a,c) and LAFP01/C (b,d) at different magnifications. (e) EDS mapping of Al, Fe, P, and O of LAFP01/C.

The morphological comparison between LFP and Al-doped LFPs at various magnifications is illustrated in Figures 1 and S1. These figures reveal that undoped LFP and Al-doped LAFPs are composed of irregularly shaped secondary particles with similar agglomeration sizes (Figure 1a and b). A closer examination at higher magnifications (Figure 1c and d) shows that the primary particles of LAFPs, approximately 150 nm in size and spherical in shape, closely resemble those of undoped LFP. Moreover, energy dispersive X-ray spectroscopy (EDS) mapping of LAFP01 (Figure 1e)demonstrates a uniform distribution of the elements Al, Fe, P, and O within the analyzed region, and its corresponding element composition, shown in Figure S2, confirms the concentration of Al.

**Figure 2:**
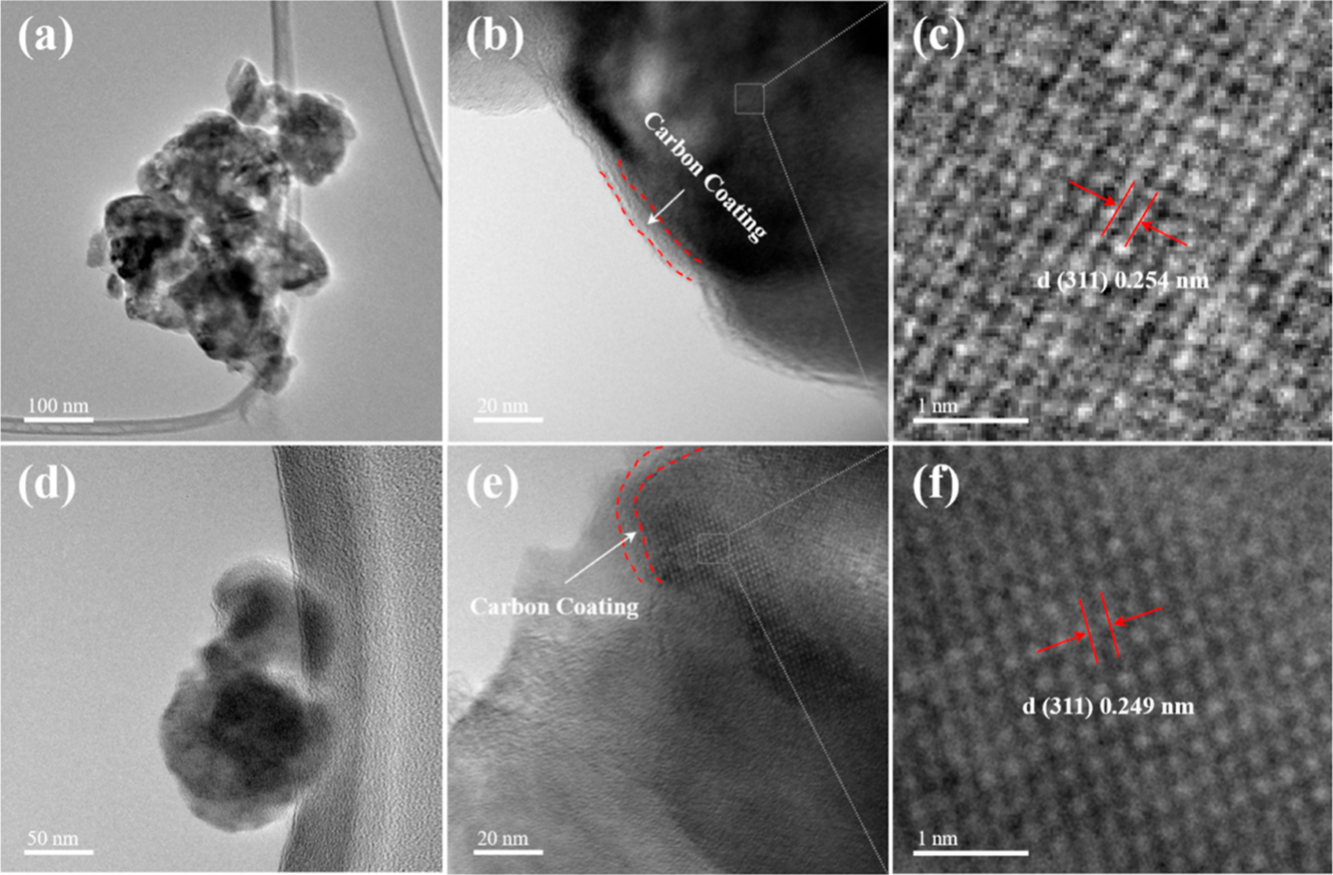
(a–c) HRTEM images of undoped LFP, (d–f) HRTEM images of LAFP01.

HRTEM images of LFP and selected LAFPs are shown in Figure 2b and e, revealing a clear, homogeneously distributed carbon coating layer with a thickness of 1–2 nm on the outer surfaces of all samples. The carbon content of each sample was quantitatively estimated using thermogravimetric analysis, as illustrated in Figure S3 and summarized in Table S2, yielding values of 6.81, 6.99, 6.78, and 7.04 wt % for LFP, LAFP01, LAFP02, and LAFP03, respectively. These results indicate that Al doping does not significantly alter the morphology of doped LFP or the formation and distribution of the carbon coating.

More importantly, Figure 2c and f displays the continuous lattice fringe with a *d*-spacing of 2.54 Å in undoped-LFP and 2.49 Å in LAFP01, corresponding to the (311) crystal plane of the LiFePO_4_ material.^17^ The reduction in d_(311)_ observed in LAFP01 provides compelling evidence for the successful incorporation of Al into LFP. This trend in *d*-spacing changes is further illustrated in the XRD measurement.

#### 3.1.2 X-ray Powder Diffraction

**Figure 3:**
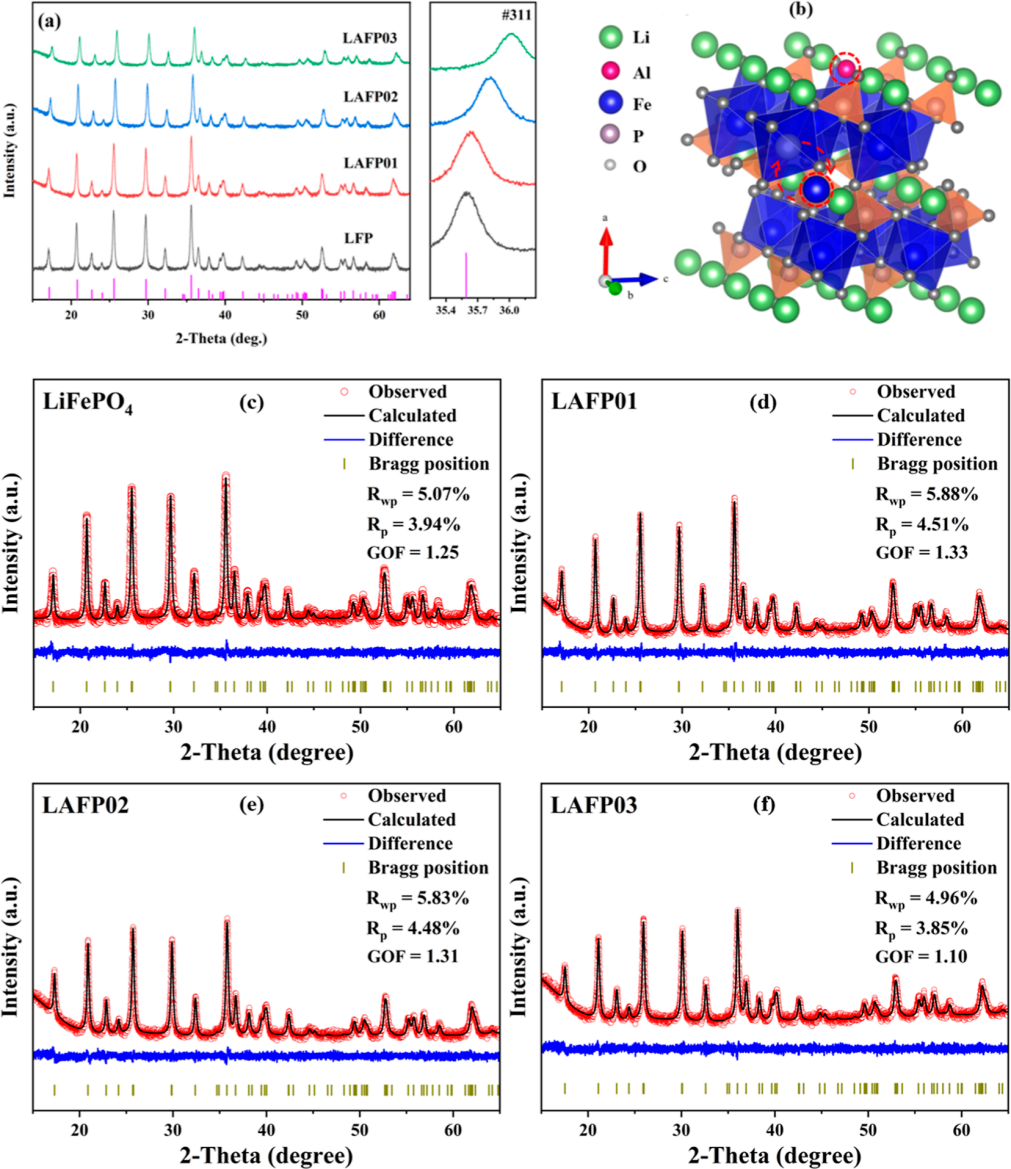
(a) XRD patterns of LFP and Al-doped LAFPs. (b) Schematic diagram of Al-doped LFP containing Fe–Li antisite defects (In the upper circled region, the red aluminum ion occupies the position of the green lithium ion at the M1 site, illustrating the presence of aluminum doping; In the lower circled region with arrows, the blue iron ion and the green lithium-ion swap their positions, leading to the formation of Fe_Li_^·^). (c–f) Reitveld refined pattern of LFP, LAFP01, LAFP02, and LAFP03.

The XRD patterns of as-synthesized LFP and LAFPs samples, alongside the standard powder diffraction file (no. 81-1173), are depicted in Figure 3. All XRD profiles exhibit a single-phase structure closely resembling the standard PDF, indexed as the orthorhombic olivine lattice in the *Pmna* space group.^7^ The sharp and narrow diffraction peaks of all samples indicate the devoid of detectable impurities such as Li_3_PO_4_,^18^ Fe_2_P,^19^ and Fe_2_P_2_O_7_.^20^ A clear and gradual rightward shift of the (311) diffraction peak in LAFPs with increasing doping content is observed in the amplified area of Figure 3a. This shift is attributed to the reduced *d*-spacing of the (311) crystal plane, as visualized in the HRTEM results.

The detailed lattice information for the as-synthesized LFP and LAFPs was obtained through Rietveld refinement (Table 1), with a goodness-of-fit (GOF) factor consistently ranging from 1 to 2 (approximately 1.3) across all profile fittings, demonstrating the high reliability of the refinement results. Given the smaller ionic radius of Al^3+^ (0.535 Å) compared to that of Li^+^ (0.76 Å), the unit cell parameters of the samples, particularly the cell volume, are consistently smaller in all LAFPs compared to that in undoped LFP. Additionally, a gradual change in lattice parameters with increasing Al doping content was also observed.

**Table 1:** Lattice Information and Fe_Li_ Defect Concentration of LFP and LAFPs Samples

lattice parameter (*Pmna*)						reliability		
sample	*a* (Å)	*b* (Å)	*c* (Å)	volume (Å^3^)	Fe_Li_ occup. (%)	*R*_wp_ (%)	*R*_p_ (%)	GOF
LFP	10.3015(3)	5.9962(1)	4.6885(1)	289.609(8)	2.04	5.07	3.94	1.25
LAFP01	10.3018(3)	5.9959(2)	4.6875(1)	289.540(9)	1.67	5.88	4.51	1.33
LAFP02	10.2958(3)	5.9944(2)	4.6907(1)	289.496(7)	2.73	5.83	4.48	1.31
LAFP03	10.2945(3)	5.9939(2)	4.6902(2)	289.405(9)	5.51	4.96	3.85	1.10

Regarding defects, Islam *et al*. identified the Li–Fe antisite defect (see Figure 3b) involving an interchange between Li ions on the M1 site and Fe ion on the M2 site, as the most favorable intrinsic defect in LiFePO_4_, primarily due to its low formation energy (0.74 eV).^21,22^ Several studies on LFP substitutions also support the prevalence of Li–Fe antisite defects in samples synthesized via hydrothermal and solid-state methods,^23−26^ and such a Li–Fe exchange leads to the creation of Li vacancies, correspondingly.^27−29^ Additionally, Axmann *et al*. highlighted the asymmetry of this Li–Fe exchange, with Fe ions on Li sites being more common than Li ions on Fe sites, attributed to the high formation energy of the latter.^25,30,31^

Building upon these findings, we carefully investigate the Fe_Li_^·^ antisite defects in both as-synthesized LFP and LAFPs (Table 1). In undoped LFP, the Fe_Li_^·^ antisite defect is found to be 2.04%, consistent with previous research reporting that the concentration of this defect should be below or around 5% for low-level substituted LFP.^23,32^ Remarkably, the LAFP01 sample demonstrates a lower Fe_Li_^·^ defect (1.67%) compared to other samples, especially undoped LFP. However, this defect noticeably increases with higher Al-doping levels, becoming more critical when the dopant concentration reaches a certain threshold (≥2%). The reduced defect content in LAFP01 can be attributed to the substitution of a limited amount of aluminum with its smaller ionic radius at the Li site. This substitution reduces the M–O (M = Li/Al) bond length within the crystal lattice, leading to a contraction of the LiO_6_ octahedron.^33,34^ Such structural shrinkage makes the formation of Li–Fe antisite defects less favorable. However, as the Al^3+^ dopant concentration increases, the size mismatch between Al^3+^ and Li^+^ becomes more pronounced, potentially destabilizing the crystal structure and thereby promoting the formation of antisite defects. In conclusion, a small amount of Al doping (up to 1%) appears to inhibit the formation of antisite defects in crystals. However, this beneficial effect is reversed when the doping concentration is increased further.

#### 3.1.3 Fourier Transform Infrared Spectroscopy

**Figure 4:**
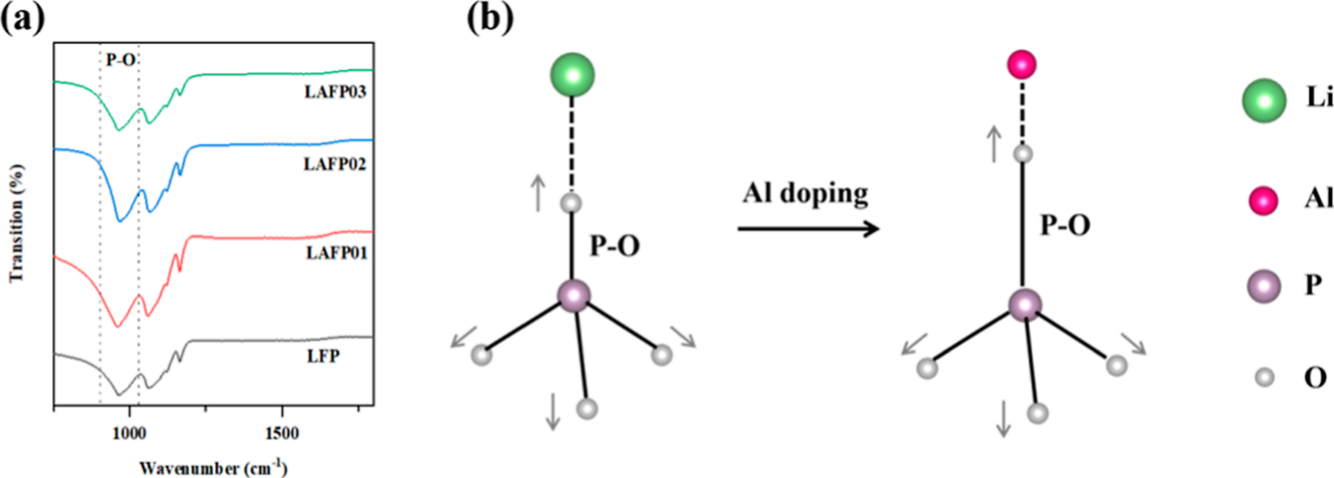
(a) FT-IR profiles of LFP and LAFPs. (b) Schematic diagram of P–O bond change of PO_4_ in LFP before and after aluminum doping.

To validate the Al-doping at Li sites within synthesized LFP samples and its impact on chemical bonds in the crystal structure, Fourier transform infrared spectroscopy (FT-IR) was employed. Extensive studies have already examined the vibrational motions of LFP and the associated absorption peak positions in bulk.^35,36^ In alignment with prior research findings,^37,38^ the detailed spectrum of LFP and LAFPs, shown in Figure 4a, indicates the absence of detectable peaks between 660 and 900 cm^–1^. This observation confirms the absence of phosphate anion impurity phases such as (P_2_O_7_)^4–^ and (P_3_O_10_)^5–^ within the crystal,^18,39,40^ affirming the high purity and well-crystallized nature of the synthesized samples.

The introduction of Al^3+^ ions into the LFP lattice typically induces changes in the chemical bonds within the crystal. The FT-IR spectra exhibit frequency bands around 1120–940 cm^–1^, associated with the symmetric stretching vibrations of P–O bonds(*v*_1_).^41−43^Figure 4b presents a schematic diagram illustrating the *v*_1_ stretching model of PO_4_ in LFP before and after doping. The theoretical substitution of Li^+^ ions by a smaller radius Al^3+^ at the 4a site leads to an expansion of the original P–O bond due to the reduced M–O (M = Li, Al) bond length. According to classical vibrational frequency principles, the P–O bond vibrational frequency *v*_1(P–O)_ can be given as
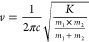
1
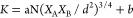
2where *c* is the light speed, *K* is the bond force constant, the term  is the reduced mass (μ), *N* is the electrostatic stress, *X*_A_ and *X*_B_ are parameters related to electronegativity involved in the bond, and *d* is the bond length. It follows that *v*_1(P–O)_ in LFP is inversely proportional to the P–O bond length *d*^3/4^. Consequently, aluminum doping in LFP is anticipated to cause expansion of the P–O bond and then result in a reduction of the *v*_1(P–O)_ vibrational frequency.

Additionally, the presence of Fe_Li_^·^ antisite defects in the LFP crystal structure can also influence the *v*_1(P–O)_ vibrational frequency by increasing it due to changes in the force constant of phosphate groups.^33,38^ The band at the 957 cm^–1^ wavenumber in the infrared absorption spectrum corresponds to the *v*_1(P–O)_ of defect-free LiFePO_4_.^44^Table 2 summarizes the *v*_1(P–O)_ vibrational frequencies of the as-synthesized LFP and LAFPs. A noticeable right shift to 964.496 cm^–1^ is observed in undoped LFP, indicating the presence of antisite defects. LAFP01 exhibits a smaller wavenumber (960.764 cm^–1^), implying a lower antisite defect content compared to the undoped material. Interestingly, a nonlinear relationship emerges between *v*_1(P–O)_ and the increasing concentration of Al doping in LAFP02 and 03. This behavior, likely influenced by the combined effects of Al doping and Fe_Li_^·^ defects, remains unclear and is beyond the scope of this study. We hope to explore this further in our future research.

**Table 2:** *v*_1(P–O)_ P–O Sym Stretching Vibrational Frequency of LFP and LAFPs

sample	P–O Sym stretching vibration (*v*_1(P–O)_) (cm^–1^)
LFP	964.496
LAFP01	960.764
LAFP02	967.384
LAFP03	964.512

#### 3.1.4 X-ray Photoelectron Spectroscopy

**Figure 5:**
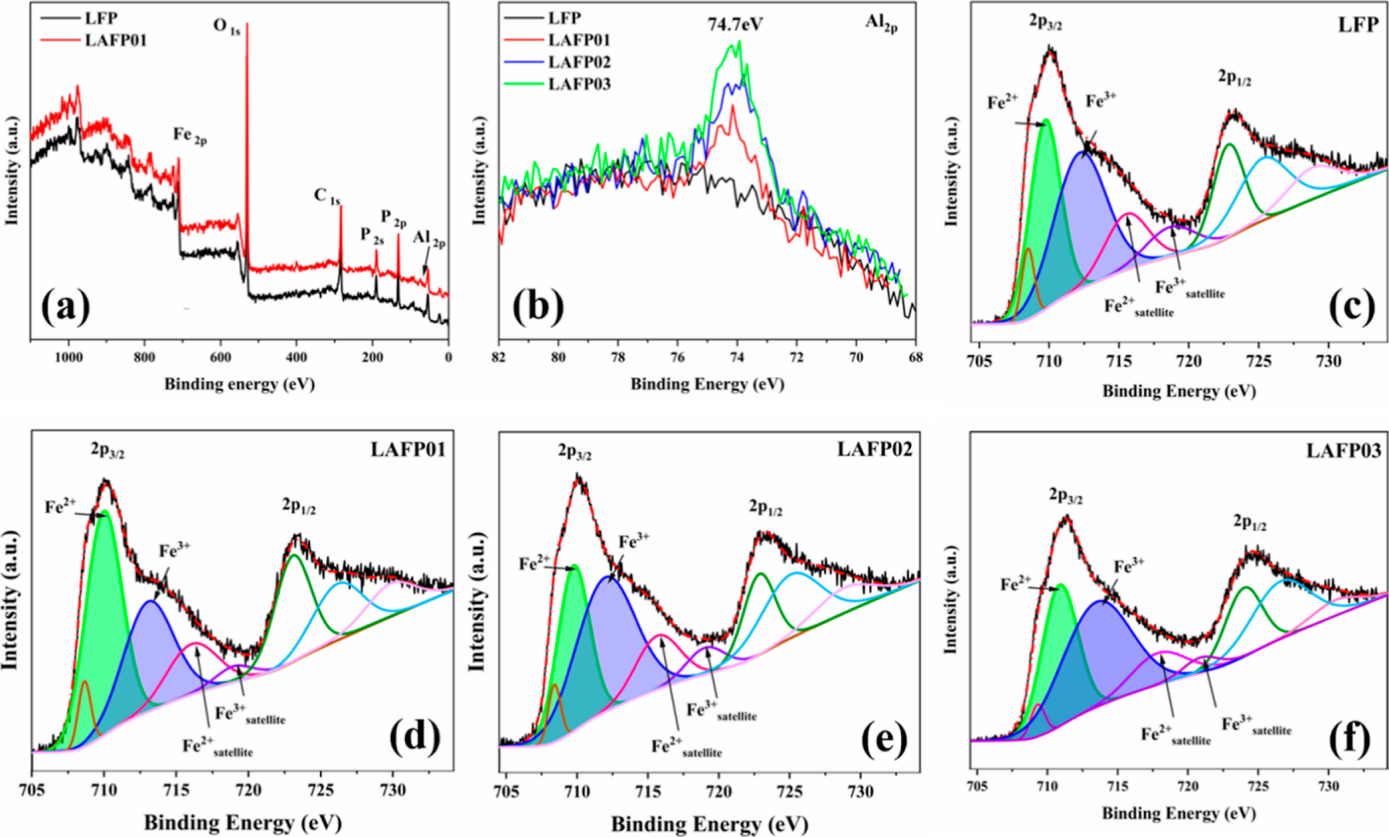
XPS spectra. (a) Survey XPS spectra of LFP and LAFP01. (b) Al 2p XPS spectra of LFP and LAFPs. (c–f) Fe 2p XPS spectra of LFP and LAFPs.

The chemical state and elemental contents of the samples were further investigated. Comparing the spectra shown in Figure 5a, the characteristic peaks of Li, P, O, Fe, and C are present in both samples, but the extra peak for Al_2p_ can be found in LAFP01. In the high-resolution XPS spectra of Al_2p_ of all samples (Figure 5b), obvious peaks at 74.7 eV with increasing intensity are observed in all LAFPs, corresponding to the rising of Al-doping content in LAFPs.^45^ More importantly, to study the lattice distortion of the LAFPs, the characterization peak of Fe 2p of the samples is analyzed and compared. Figure 5c–f displays the high-resolution Fe_2p_ spectra, composed of separated regions corresponding to Fe_2p3/2_ and Fe_2p1/2_ due to spin–orbit splitting.^46^ Furthermore, the separated region can be deconvoluted into several peaks that are related to the main core-level peaks for Fe^2+^/Fe^3+^ and their associated satellite peak. To conduct a semiquantitative analysis of iron (Fe) content with varying valence states, peak fitting was employed, and the results are shown in Table 3.

**Table 3:** Fitted Peak Quantification of High-Resolution Fe XPS Spectra of LFP and LAFPs

	analytical result					
	Fe_2p3/2_		Fe_2p1/2_			
sample	Fe^2+^	Fe^3+^	Fe^2+^	Fe^3+^	Fe^3+^ area ratio % (%)	reliabilityChi^2^
LFP	709.72	712.19	722.82	725.29	27.76	1.43
LAFP01	709.97	713.07	723.07	726.17	21.26	1.21
LAFP02	709.74	711.97	722.84	725.07	31.46	1.17
LAFP03	709.31	713.49	723.42	726.59	32.63	1.45

Consistently, the Fe 2p spectra for both undoped LFP and LAFPs exhibit similar subpeaks with matched binding energies, approximately 710 eV for the Fe^2+^ main peak (with its satellite at around 715 eV) and approximately 712 eV for the Fe^3+^ main peak (with its satellite at about 718 eV).^46−48^ Moreover, there is a decrease in Fe^3+^ percentage in LAFP01 compared to that in undoped LFP (light blue area under the curve), declining from 27.76% to 21.26%. In contrast, the Fe^3+^ content in LAFP02 not only exceeds that of undoped LFP but also continues to rise as the Al-doping content increases, a trend further evidenced when comparing LAFP02 to LAFP03.

This correlation between the Fe^3+^ concentration in LFP and its aluminum-doped counterparts (LAFP01) aligns closely with the variations in Fe_Li_^·^ antisite defects identified through our previous analysis. Specifically, a notable reduction in both Fe_Li_^·^ antisite defects (XRD) and Fe^3+^ concentration (XPS) was observed in LAFP01 when compared to undoped LFP. We attribute this reduction to the limited presence of Fe_Li_^·^ antisite defects. These defects elevate the oxidation state of Fe at the antisite position, resulting in a higher percentage of Fe^3+^ compared to the average Fe-oxidation state (+2) in the bulk material.^49^ Therefore, a lower Fe^3+^ concentration indicates fewer Fe_Li_^·^ antisite defects.

### 3.2 Electrochemical Performance Evaluation

**Figure 6:**
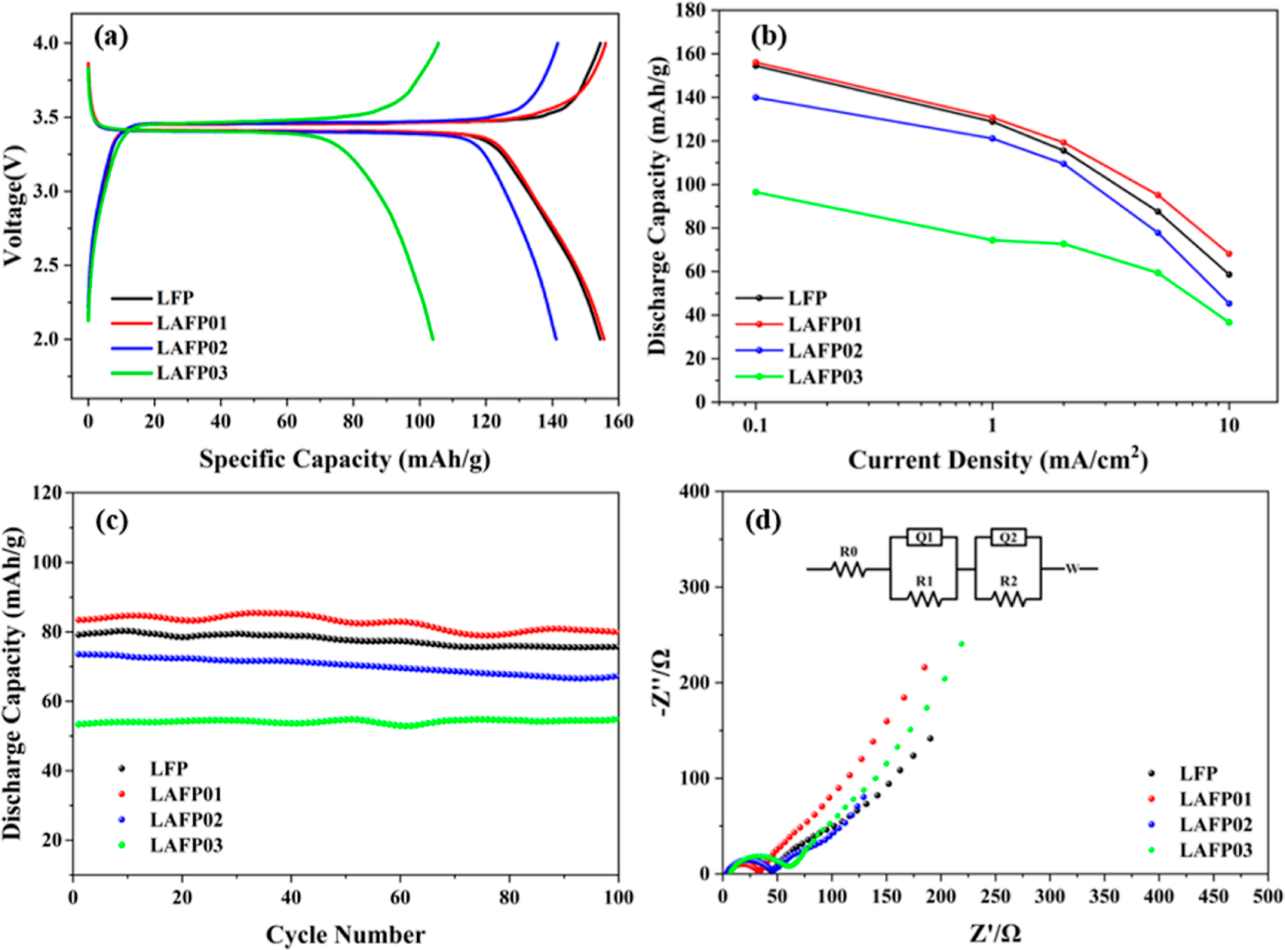
Electrochemical properties. (a) voltage–capacity curves at 0.1 C. (b) Ragone plot for as-synthesized LFP and LAFPs. (c) Long cycling stabilities at 5 C. (d) EIS plots and their equivalent circuit.

The galvanostatic cycling profile of as-synthesized LFP and LAFPs at 0.1 C is shown in Figure 6a. In all cases, we observe voltage plateaus at approximately 3.45 V, a characteristic feature associated with the phase transition between LiFePO_4_ and FePO_4_ during lithium intercalation and deintercalation.^34,50^ Remarkably, LAFP01 exhibits an exceptional discharge capacity of 155.6 mA h/g at 0.1 C, surpassing those of the undoped LFP and the rest of LAFPs (including LAFP02 and LAFP03), which have capacities of 154.4, 141.2, and 104.1 mA h/g, respectively.

Figure 6b presents a comparison of the rate performance of samples at charge and discharge current densities from 0.1 to 10 C. Notably, LAFP01 consistently maintains the highest discharge capacity across all C-rates. Moreover, as the current density increases, the delivered capacity of undoped LFP decreases more visibly than that of LAFP01, highlighting the superiority of its performance. Again, the limited Fe_Li_^·^ concentration in LAFP01 could be the reason for this, as the ion diffusion within its crystal lattice faces fewer obstructions compared to other samples.^23^

To further validate our findings, we subjected the prepared samples to long cycling tests at 5 C. The result, illustrated in Figure 6c, aligns well with our expectations. Benefiting from the less blocking effect of low Fe_Li_^·^, LAFP01 exhibits the highest specific capacity, particularly when compared to undoped LFP. Importantly, the capacity retention of undoped LFP and LAFPs shows no significant difference within 100 cycles, suggesting that substituting a small amount of lithium with Al does not noticeably affect the cycling stability of LFP materials.

To quantitively confirm the effect of Fe_Li_^·^ defects on the improved electrochemical performance of LAFP01, the ion diffusion rate inside of the material is tested via the EIS method. The Nyquist plots for LFP, LAFP01, LAFP02, and LAFP03 are presented in Figure 6d. The EIS profiles of all samples feature a semicircle in the high- to middle-frequency region and a slash in the low-frequency region, which are commonly associated with the charge transfer resistance *R*_ct_ and Warburg impedance *W*, respectively. Therefore, we fit the EIS profiles to an equivalent circuit, which includes an Ohmic resistance *R*_0_, an *R*_1_||*Q*_1_ element representing the lithium-ion migration in SEI and surface heterogeneous reactions,^51^ an *R*_2_||*Q*_2_ element corresponding to the film solution interface, and a Warburg element for ion diffusion.^50,52−55^ The Warburg impendence is linked to the Li^+^ ion diffusion coefficient *D*_Li^+^_ through the following equation^56^
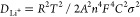
3where σ is the Warburg factor extracted from fitting. The Warburg factor and diffusion coefficient of lithium ions (*D*_Li^+^_) calculated from eq 3 are summarized in Figure S4 and Table 4.

**Table 4:** Extracted Warburg Factor and Calculated Diffusion Coefficient of As-Prepared Samples

sample	σ_w_ (Ω·s^–1/2^)	*D*_Li^+^_ (cm^2^·s^–1^)
LFP	32.98	2.75 × 10^–11^
LAFP01	31.08	3.10 × 10^–11^
LAFP02	45.68	1.43 × 10^–11^
LAFP03	59.77	8.37 × 10^–12^

As doping content *x* increases from 0 (undoped) to 0.03 (doped) in Li_1–3*x*_Al_*x*_FePO_4_, the *D*_Li^+^_ of samples initially rises, followed by a decrease. More importantly, LAFP01 (*x* = 0.01) contains the highest value of 3.10 × 10^–11^, which signifies that LAFP01 (1% doping) has the highest lithium-ion diffusion rate, which correlates with its observed superior discharge capacity.

**Figure 7:**
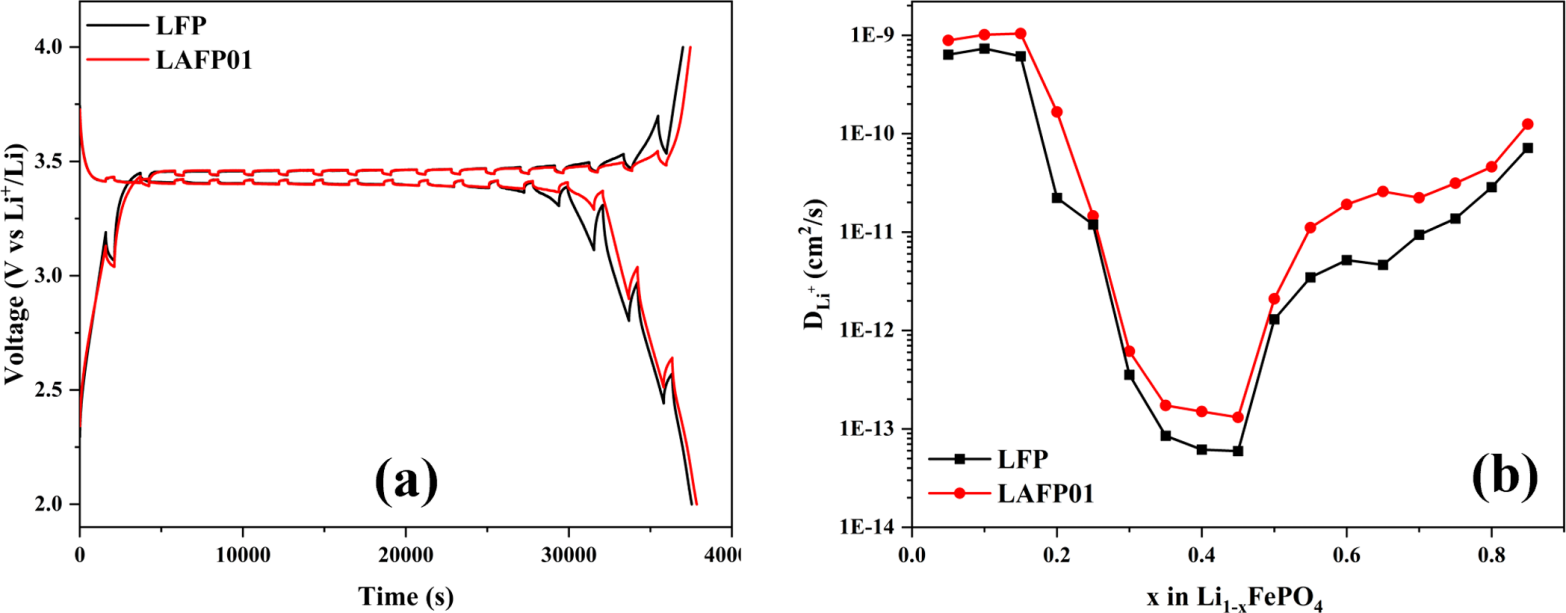
Galvanostatic Intermittent Titration Technique (GITT) tests. (a) GITT curve of undoped LFP and LAFP01 during charge and discharge; (b) lithium-ion diffusion coefficient calculated from the charge curve of undoped LFP and LAFP01.

Galvanostatic Intermittent Titration Technique (GITT) measurement of undoped LFP and LAFP01 was also conducted after EIS, and the charge/discharge curve of samples for the third cycle was obtained by cycling the samples at a galvanostatic current of 0.1 C for 30 min, followed by an open-circuit relaxation for 10 min, under a voltage range from 2 to 4 V (Figure 7a). The diffusion coefficient of lithium-ion *D*_Li^+^_ can be calculated based on the following equation^57^
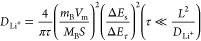
4

The *D*_Li^+^_ as a function of the stoichiometry *x* in Li_1–*x*_FePO_4_/Li_1–3*x*_FePO_4_ is shown in Figure 7b. During the GITT charge, LAFP01 has a Li^+^ diffusion coefficient, ranging from 1.04 × 10^–9^ to 1.31 × 10^–13^ cm^2^ s^–1^, which is consistently higher than that of undoped LFP from 7.32 × 10^–10^ to 5.95 × 10^–14^. The highly consistent results from both EIS and GITT enable us to conclude that a 1% Al doping in LFP is beneficial, as it creates the least Fe_Li_^·^ antisite defect, resulting in a higher diffusion rate that improves product performance, especially during fast charge and discharge.

## 4 Conclusions

The synthesis of pure LFP/C and Li_1–3*x*_Al_*x*_FePO_4_/C (where *x* = 0.1, 0.2, and 0.3, denoting the molar percentage of dopant) was accomplished through a one-step high-temperature solid-state reaction. Employing a combination of X-ray diffraction (XRD) with Rietveld refinement and Fourier-transform infrared spectroscopy (FT-IR), we thoroughly examine the structural implications of aluminum doping on LFP. We confirm the effective incorporation of Al^3+^ into the Li site (M1) to form a solid solution, with only marginal alterations to the lattice parameters. Furthermore, in terms of crystal defects, it was observed that a low level of aluminum doping (≤1%) suppresses the emergence of Fe_Li_^·^ antisite defects. However, this beneficial effect is negated by a further increase of doping concentration (over 1%); the higher defect content of LAFP02 to LAFP03 than that of undoped LFP suggests that excessive Al doping may exacerbate cationic disorder within the LFP crystal structure.

Electrochemically, LAFP01, characterized by a 1% aluminum doping, exhibits the highest discharge capacity across various ranges of C-rate, achieving 155.6 mA h/g at 0.1 C and 86 mA h/g at 5 C. Combining the findings from structural characterization, we suggest that the suppression of Fe_Li_^·^ antisite defects, potentially induced by Al doping, plays a role in the performance enhancement of doped LFP. Importantly, the advantage of LAFP01 on electrochemical performance becomes increasingly more pronounced with rising charge and discharge rates. This trend indicates that an appropriate Al-doping level is beneficial and could contribute more to improving the electrochemical performance of LFP under rapid charge and discharge conditions. The established correlation between aluminum doping and the properties of doped LFP presented in this study offers valuable insights for managing aluminum impurity levels in battery recycling within an industrial regime.

## Supporting Information Available

SEM image of samples; EDS element composition spectrum of LAFP01; TG curve of synthesized LFP/C and LAFPs/C; Correlation between *D*_Li^+^_ and the aluminum doping content; ICP-OES of LFP and LAFPs; Carbon contents of synthesized LFP/C and LAFPs/C (PDF)
