# Supplementary material for: Doping LiFePO4 with Al3+: Suppression of Anti-Site Defects and Implications for Battery Recycling
Source: ACS Omega. 2025 Jan 6;10(1):1293–302. doi: 10.1021/acsomega.4c08870 (PMC11740143; doi:10.1021/acsomega.4c08870)
Supplement: Supplementary file 1 — ao4c08870_si_001.pdf [file ao4c08870_si_001.pdf]

Supporting Information

Doping $LiFePO_4$ with Al^{3+}: Suppression of Anti-Site Defects and Implications for Battery Recycling

Yunhao Xiao,* Zihang Zhao, Qipeng Zhang, and Rui Qiao*

Department of Mechanical Engineering, Virginia Tech, Blacksburg, VA

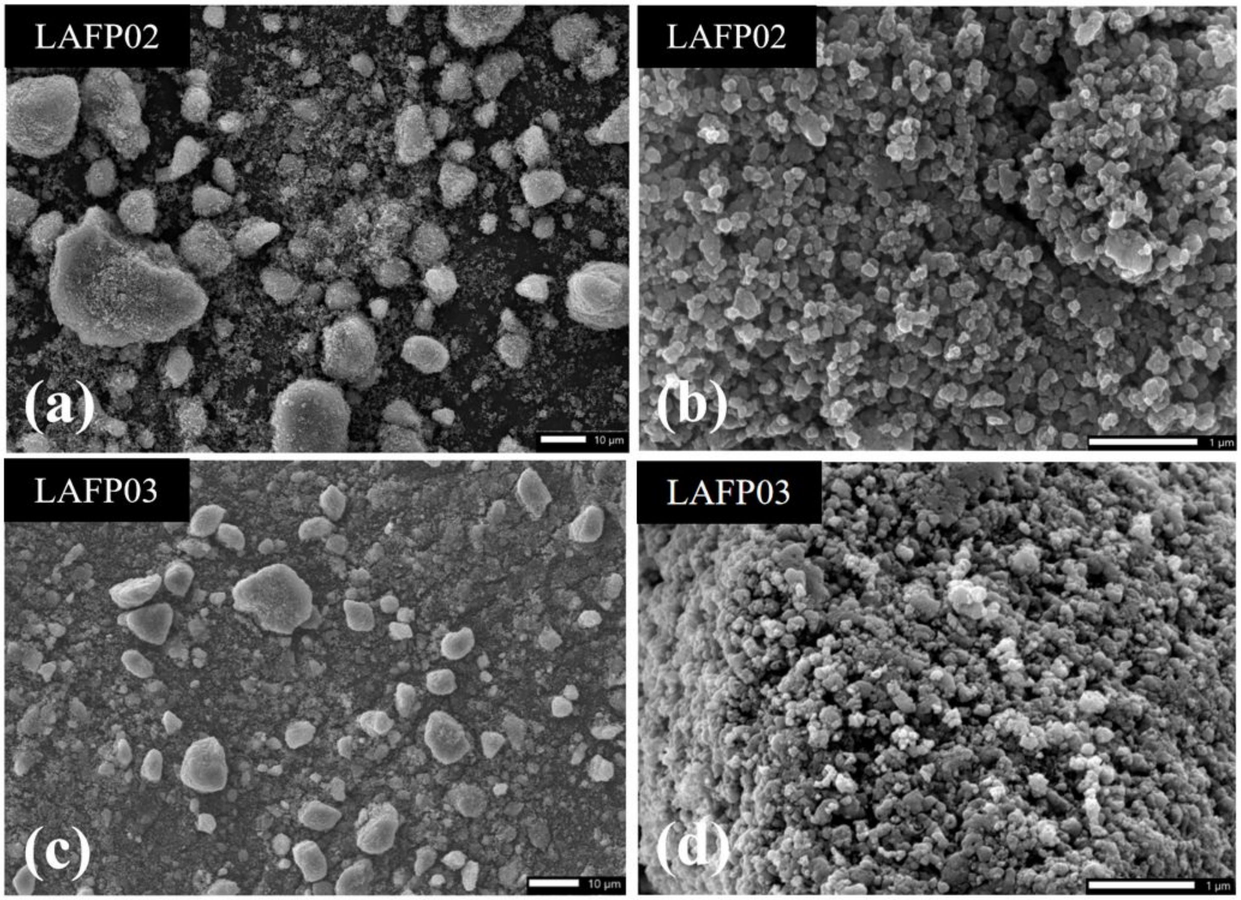

Figure S1. SEM image of samples at different magnifications. (a)(b) LAFP02. (c)(d) LAFP03.

* To whom correspondence should be addressed. Emails: xyunhao@vt.edu (Y.X.), ruiqiao@vt.edu (R.Q.).

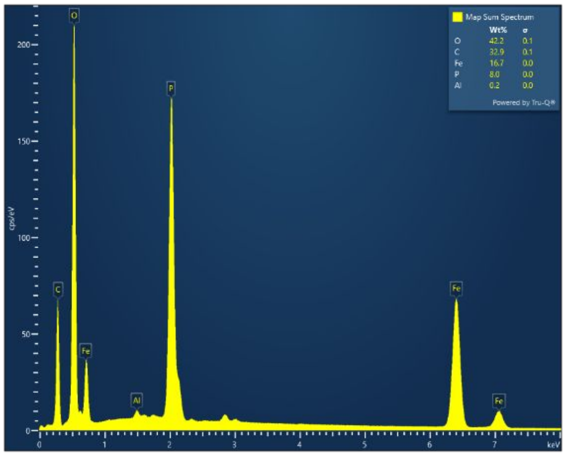

Figure S2. EDS element composition spectrum of LAFP01.

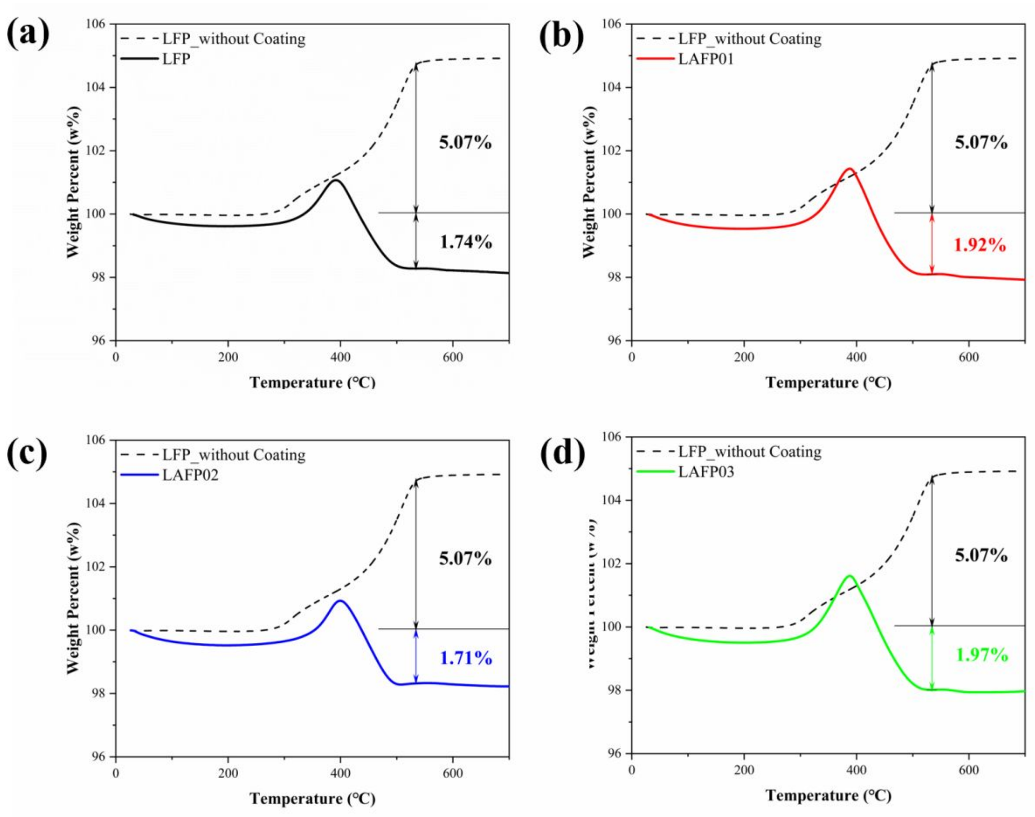

Figure S3. The TG curve of synthesized LAFPs/C tested in an oxygen atmosphere. (a) LFP. (b) LAFP01. (c) LAFP02. (d) LAFP03. The LFP and carbon oxidation is completed when the temperature reaches 500℃, The black dot line in each figure is the TG curve of pure LFP without carbon coating, which serves as a benchmark for LAFPs/C that corresponds to a theoretical weight gain of 5.07%[1]. The actual amount of carbon content in as-synthesized LAFPs/C is the difference between the theoretical weight gain of pure LFP and the observed weight change (gain or loss) of LAFPs/C.

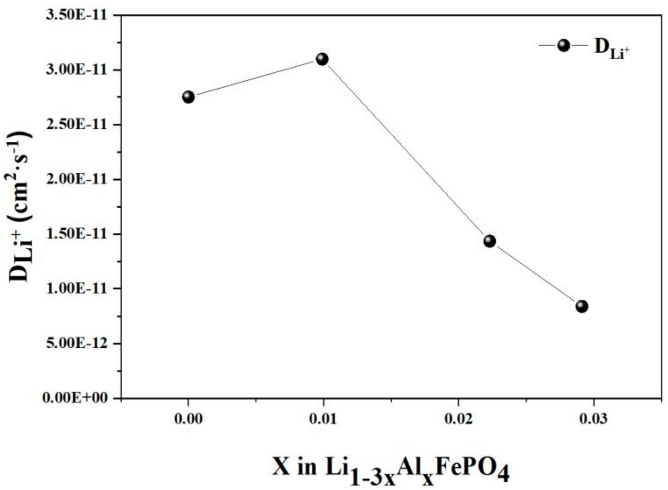

Figure S4. Correlation between D_{Li^+} and the content of aluminum doping x in LFP ($0< x < 0.03$).

Table S1. ICP-OES of LFP and LAFPs.

Contents (ppb)	Al	Cr	Ni	Co	Cu
LFP	2.8	0.3	1.5	0.4	0.3
LAFP01	13.3	0.3	1.5	0.4	0.4
LAFP02	27.2	0.4	1.4	0.4	0.4
LAFP03	47.2	0.7	1.4	0.4	0.8

Table S2. The carbon contents of synthesized LAFPs estimiated by thermogravimetric analysis.

Samples	Carbon Content (wt%)
LFP	6.81
LAFP01	6.99
LAFP02	6.78
LAFP03	7.04

References

(1) Zhao, J.-W.; Zhao, S.-X.; Wu, X.; Cheng, H.-M.; Nan, C.-W. Double Role of Silicon in Improving the Rate Performance of LiFePO4 Cathode Materials. *J. Alloys Compd.* **2017**, *699*, 849–855. https://doi.org/10.1016/j.jallcom.2016.12.430.
